# Social media in myositis care – an exploratory mixed-methods study among myositis patients (SociMyo)

**DOI:** 10.1007/s00296-025-05903-6

**Published:** 2025-06-04

**Authors:** Katharina Boy, Niklas Ohm, Susann May, Greta Nordmann, Lynn Wilson, Johannes Knitza, Martin Heinze, Latika Gupta, Felix Muehlensiepen

**Affiliations:** 1 Brandenburg Medical School Theodor Fontane, Center for Health Services Research, Faculty of Health Sciences Brandenburg, Rüdersdorf bei Berlin, Berlin, Germany; 2 Myositis Support and Understanding (MSU), Lincoln, Delaware, United States of America; 3 Institute for Digital Medicine, University Hospital of Giessen and Marburg, Philipps-University Marburg, Marburg, Germany; 4 Department of Psychiatry and Psychotherapy, Immanuel Klinik Rüdersdorf, Rüdersdorf bei Berlin, Berlin, Germany; 5 School of Infection, Inflammation and Immunology, College of Medicine and Health, University of Birmingham, Birmingham, UK; 6 Division of Musculoskeletal and Dermatological Sciences, Centre for Musculoskeletal Research, School of Biological Sciences, The University of Manchester, Manchester, UK; 7 Francis Crick Institute, London, UK; 8 Department of Rheumatology, Royal Wolverhampton Hospitals NHS Trust, Wolverhampton, UK; 9 Univ. Grenoble Alpes, AGEIS, Grenoble, France; 10 Center for Health Services Research, Brandenburg Medical School Theodor Fontane, Seebad 82/83 Rüdersdorf bei Berlin, 15562 Berlin, Germany

**Keywords:** Myositis, Autoimmune diseases, Social media, Health services research, Rare diseases, Telemedicine

## Abstract

Myositis is a rare autoimmune disease primarily affecting muscles, with potential involvement of the skin, heart, and lungs. Patients often experience delays in diagnosis, lack of adequate information, and limited support for disease management. Social media has emerged as a valuable tool to address these gaps by facilitating information exchange, peer support, and community building. However, its role in myositis care is not yet well understood. This study aims to explore how myositis patients use social media, focusing on shared content, perceived benefits and challenges, and the overall impact on disease management and emotional well-being. A mixed-methods approach was applied, including semi-structured interviews with 11 patients and a netnographic analysis of social media group dedicated to myositis care. Data were analyzed using Kuckartz’s structured qualitative content analysis, with coding performed inductively, to identify key themes. Four key themes emerged: (I) Social media as a global platform for sharing experiential knowledge, particularly on symptom management, medication side effects, and coping strategies. (II) Peer support fostering a sense of belonging, emotional exchange, and mutual encouragement through structured discussions and community-driven moderation. (III) Perceived benefits, such as real-time access to patient-driven insights, shared decision-making support, and enhanced communication with healthcare providers. (IV) Perceived drawbacks, including misinformation, privacy concerns, and the absence of professional medical input. Participants emphasized the need for expert involvement to improve content reliability, while also valuing the autonomy and emotional support within these communities. Social media platforms, particularly closed groups, provide a complementary avenue to traditional care by offering support and knowledge exchange. To maximize their potential, privacy concerns and the integration of professional guidance must be addressed.

**Supplementary Information:**

The online version contains supplementary material available at 10.1007/s00296-025-05903-6.

## Introduction

Inflammatory diseases of the skeletal muscles are serious and often debilitating conditions that significantly impair quality of life. In addition to muscle weakness, these diseases frequently involve other organs, such as the heart, lungs, and esophagus, leading to symptoms like dyspnea or dysphagia [1]. Among these conditions, myositis poses a particularly significant and persistent challenge for patients, their families and friends, and healthcare providers [2]. The rarity of these diseases, combined with their complexity and often nonspecific symptoms, frequently lead to substantial diagnostic delays, further complicating timely and effective management [3].

Patients require accurate and comprehensive information about their condition during the diagnostic process and even after a diagnosis has been made [3]. Patients face challenges in identifying effective self-management tools and frequently experience unmet needs regarding self-management strategies, which can further complicate their care [4]. Despite the increasing availability of digital health solutions, their potential to support self-management in idiopathic inflammatory myopathies remains largely untapped [5]. Yet, workforce shortages in healthcare often limit the time available for in-depth consultations, leaving many patients feeling inadequately informed [6]. Additionally, research in rheumatic and musculoskeletal diseases (RMD) care has shown that healthcare providers frequently underestimate the scope of patients’ informational needs [7]. Consequently, many patients turn to the internet to address their need for information. This approach, however, carries significant risks, as online health content is mostly unverified or even inaccurate [8, 9]. Studies assessing the quality of online content, including YouTube videos on inflammatory myositis, highlight considerable variability in reliability and educational value for both patients and physicians [10]. In fact, in an international survey among patients with antisynthetase syndrome, a rare autoimmune condition affecting muscles, lungs and the musculoskeletal system, only less than one-third (30.1%) of the participants reported to know useful online information sources related to antisynthetase syndrome [3]. Even when patients do find information, there is typically no follow-up mechanism to ensure that they understand and appropriately act on it [11]. This lack of guidance not only jeopardizes the quality of patient care but also underscores the urgent need for verified, accessible, and patient-centered resources to bridge the gap in informational support.

In the last decade, social media platforms have emerged as a prominent source of health information and peer support. Unlike static online resources, these platforms enable dynamic interactions, allowing patients with chronic conditions to exchange experiences, seek advice, and build supportive communities [12]. Despite their growing relevance, the role of social media in addressing the informational and emotional needs of myositis patients—and its broader impact on disease management—remains poorly understood. A deeper exploration of this potential could reveal innovative ways to bridge existing gaps in patient care and enhance support systems. Large-scale patient-centered initiatives, such as the COVAD study, emphasize the importance of integrating patient perspectives into research and healthcare design, underscoring the potential of social media in improving patient engagement [13].

This exploratory qualitative study aimed to examine how patients with myositis use social media, involving the types of content they share, the perceived benefits and challenges of using social media in myositis care, and its overall role in providing support and enhancing care from the patients’ perspective.

## Methods

To explore patient experiences with social media in myositis care, qualitative telephone interviews were conducted alongside a netnographic analysis of a dedicated myositis-focused social media group. Integrating these methods allowed us to capture distinct yet complementary perspectives. While the qualitative interviews offered detailed individual insights into patient experiences, the netnographic approach provided a broader view of collective interactions and community discussions. This methodological triangulation yielded richer results than any single method could independently.

Data were collected between February and March 2024. Participants were recruited through international patient organizations groups, utilizing purposive and snowball sampling methods [14]. Our sampling strategy prioritized diversity in age, gender, and professional background. In total, 11 individuals participated in the interviews, without receiving financial compensation. The semi-structured interview guide (available in Supplemental Material) was specifically developed to explore patient experiences related to social media use in managing myositis. The guide was developed based on previous qualitative studies on chronic disease management and refined in collaboration with our research team. It comprised open-ended questions addressing four primary themes: social media usage behaviours, thematic content specific to myositis care, perceived advantages, and potential drawbacks. Initial exploratory questions were supplemented by targeted follow-up queries for deeper exploration. Two pilot interviews confirmed the appropriateness of the guide, necessitating no adjustments. Demographic data including gender, age, myositis diagnosis, employment status and country of residence were collected to provide contextual depth to the findings.

Qualitative analysis was carried out collaboratively by two MD students (KB and NO) and two health services researchers (SM and FM) using the structured qualitative content analysis method described by Kuckartz [15], supported by MAXQDA software (Verbi GmbH). Nurses or other healthcare professionals did not participate in data collection or analysis. After transcribing the audio recordings, the analysis started with a detailed review of the transcripts, followed by independent coding by three researchers (KB, SM, FM). Categories were inductively derived from the transcripts to develop a comprehensive coding framework, which was then systematically applied to the full dataset. Representative quotations illustrating key findings were selected and integrated into the manuscript. The manuscript adheres to the Consolidated Criteria for Reporting Qualitative Research (COREQ) guidelines (Supplemental Material) [16].During the qualitative interviews, the study team was invited to a private social media group dedicated to patients with myositis and their carers. Field notes were collected during March 2024 on relevant topics, adhering to ethical and data protection guidelines, with all observations anonymized and no personal data recorded. Prior to publication, all collected data underwent thorough review by the Data Protection Officer to ensure complete compliance with privacy regulations and to safeguard the confidentiality of all participants involved in this sensitive research context.

Netnography, as a method, facilitated the analysis of social interactions and cultural patterns within this online community [17]. The analysis included a comprehensive review of all posts ever shared in the group from its inception to the end of the observation period. In line with ethical guidelines and the access conditions stipulated by the group administrators, we adopted a purely observational role and did not actively participate in discussions, in order to respect the privacy and autonomy of the group members. The analysis focused on examining the themes of posts, the structural organization of the group, recurring practices (rituals), the language and symbols used by members. Special attention was given to the sharing of current research findings and the exchange of experiential knowledge. Posts were systematically reviewed and categorized based on their relevance to the research questions. An inductive approach was applied to identify patterns and themes emerging directly from the data allowing for an open exploration of the group dynamics and shared experiences.

The research methods form a triangulation. The insights from the interviews and the results of the netnographic analysis complement each other by offering different interconnected perspectives on the role of social media. By integrating these complementary findings, the study provides a comprehensive understanding of how social media facilitates information sharing, emotional support and the dissemination of experiential and research-based knowledge. This approach is consistent with the principles of integration in mixed-methods research and emphasises the added value of synthesising qualitative findings from different data sources [18].

## Results

### Qualitative interviews

Mean age of interviewed patients was 55 (range: 31–78) years, see Table 1. Overall, 10/11 (91%) of patients were female. Patients reported diverse occupational backgrounds and countries of residence. All patients had a myositis diagnosis. The interviews lasted between 21 and 52 (mean 29) minutes. The patients reported using various social media platforms, with Facebook being the most widely utilized among the sample.

*‘And so for me*,* Instagram is more for creative*,* right. Facebook is more for community. And they have kind of like the platform to kind of do it. And I’m not big on the… I don’t really do Twitter*,* X*,* I don’t do YouTube. Like I pretty much keep it to Facebook because you have different rooms and groups and privacy. ‘ (P 5*,* pos. 21)*.

Patients highlighted Facebook’s structured community features and privacy settings as key reasons for choosing this platform over others.

**Table 1:** Participant characteristics

Patient	Age (years)	Gender	Diagnosis since	Occupation	Residency	Used Social Media Platforms
**1**	31	female	1 year ago	Chemist	Italy	Facebook Instagram YouTube
**2**	73	female	13 years ago	Pensioner	Australia	Facebook
**3**	32	female	13 years ago	Commercial employee	USA	Facebook Instagram
**4**	59	female	6 years ago	Pensioner	UK	Facebook Instagram Twitter/X
**5**	47	female	5 years ago	Food Scientist	Costa Rica	Facebook
**6**	57	female	10 years ago	Pensioner	USA	Facebook
**7**	50	male	8 years ago	Pensioner	USA	Facebook Instagram
**8**	66	female	7 years ago	Pensioner	USA	Facebook
**9**	72	female	3 years ago	Pensioner	USA	Facebook
**10**	47	female	18 years ago	Scientist	Switzerland	Facebook Twitter/X
**11**	78	female	9 years ago	Pensioner	USA	Facebook

The qualitative analysis of the interview data generated four themes: (I) Social media as a global platform for sharing information and experiential knowledge, (II) The role of social media in fostering peer support and belonging among patients, (III) Perceived benefits of social media in myositis care, (IV) Perceived drawbacks of social media in myositis care.

### Social media as a global platform for sharing information and experiential knowledge

Patients with myositis report significant challenges in finding reliable information about their disease. The lack of accessible resources and uncertainty where to find reliable resources of information, left some feeling isolated and unsure about their diagnosis and treatment options.

*‘When I tried to find information about myositis back in 2014*,* there really wasn’t much. I mean*,* there’s more research being done now*,* but back then there really wasn’t much. And the first thing my doctor said was*, *‘Don’t Google it’.’ (P 5 pos. 39)*.

*‘Back in 2017*,* you know*,* there was very little available on Google. And*,* you know*,* many of the studies*,* half the people died before the end of the study. You know*,* so it was just not a happy time.’ (P 11*,* pos. 7)*.

Patients highlight that social media offers the possibility to connect worldwide and it is not bound to time zones.

*‘And*,* you know*,* we’re on different time zones*,* right. And so*,* it’s the craziest thing*,* every night at 3 o’clock*,* I was wide awake. It’s the side effects of the medication*,* issues with sleeping. I started messaging with someone who was motivating me*,* you know and I’m thinking - oh my goodness*,* I have a friend abroad. Like*,* it opens you up to the world.I had people I knew that were from other countries. But now*,* I’m talking to people in all these other countries.And it’s great. Like that’s one of the positive things of social media. And no matter what country you’re in*,* myositis is myositis*,* right. I mean*,* we’re all the same. It’s just we live in a different place*,* we speak with a different accent*,* right. We use different words. So you’re actually also helping*,* other people in countries that do not have the same level of medical care. Like*,* it really is a devastating disease*,* but social media allows you to have a lifeline. And that’s to me what social media is supposed to be about. It’s supposed to be about community.’ (P 5, pos. 33–37).*

Patients frequently emphasize that peer-to-peer support is an essential resource for managing chronic conditions. From their perspective, fellow patients offer invaluable experiential knowledge that goes beyond clinical treatment. This peer support helps bridge the gap between medical interventions and the practical realities of living with a chronic illness, providing insights that are deeply rooted in shared experiences.

*‘I think that other patients can sometimes provide better information than a physician*,* because a physician only sees the medical side of an illness*,* but a patient can give you all the information about how to live with the problems associated with the disease.’ (P 3*,* pos. 41)*.

### The role of social media in fostering peer support and belonging among Myositis patients

Patients highlighted the significant role of social media in sharing experiences and providing support in managing chronic conditions. They reported that peer-driven support on these platforms not only helps others avoid common pitfalls but also creates a sense of belonging and mutual understanding within the community. This type of support, facilitated by social media, was seen as a valuable complement to traditional medical care, enhancing patient well-being and strengthening interpersonal connections.

*‘I share my experiences to help others avoid some of the mistakes I made at the beginning. And because they helped me a lot in turn. And so I want to be useful too.’ (P 3*,* pos. 39)*.

*’If someone writes about a low point or an acute episode on Facebook*,* I’ll leave a like*,* a heart*,* or write something like: Hey*,* I wish you lots of strength. feel free to get in touch - I just want to offer a little support and simply say: I read it*,* I notice you*,* you are not alone.’ (P5*,* pos. 27)*.

Patients emphasized that exchanging information on social media is more than just sharing facts but about creating a personal connection. This sense of connection makes the information more relatable and meaningful, fostering a sense of trust and community among users. Information shared on social media are considered as reliable.

*‘It’s about the personal connection. The personal connection with the information*,* not just the information itself.’ (P 4*,* pos. 22)*.

*‘I haven’t seen anything recently that really makes me worried. I think*,* most of the information that I’ve seen on social media*,* on the sites that I’m familiar with the information so far I’ve seen is not too bad. There’s not been anything that I’ve had to write to them to say*,* can you please remove that or that’s not true. And I will do that if it’s something that is not useful for the public information. So far it’s been quite well moderated.’ (P 10*,* pos. 37)*.

### Perceived benefits of social media in myositis care

**Fig. 1:**
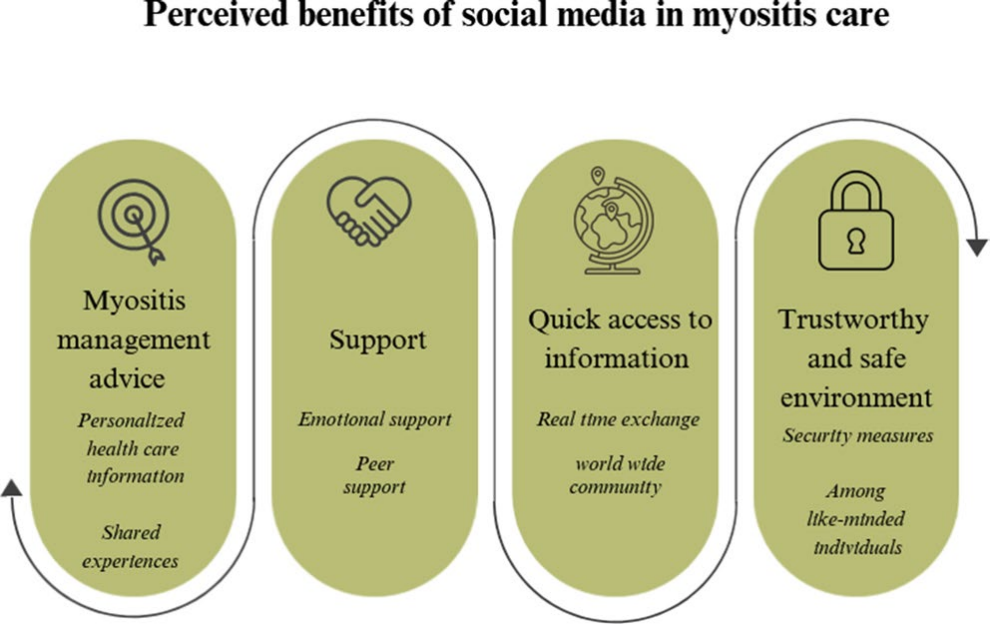
Perceived benefits of social media in myositis care, as reported in the interviews

Patients reported that social media groups offer numerous benefits (Fig. 1), including support, shared experiences, and practical advice. Social media facilitates peer support and the sharing of experiential knowledge, enabling patients to access diverse viewpoints and practical insights into managing their condition. The accessibility of these platforms allows for immediate, international real-time exchange, which is especially important for those managing complex and rare conditions. Furthermore, the emotional support offered by peers helps alleviate feelings of isolation, providing a sense of solidarity and understanding.

To ensure a safe and supportive environment, social media groups often implement security measures, such as screening questions, to verify that only genuine participants are accepted. These measures play a crucial role in creating a trustworthy space where patients can receive support, share experiences, and access practical advice.

### Perceived drawbacks of social media in myositis care

**Fig. 2:**
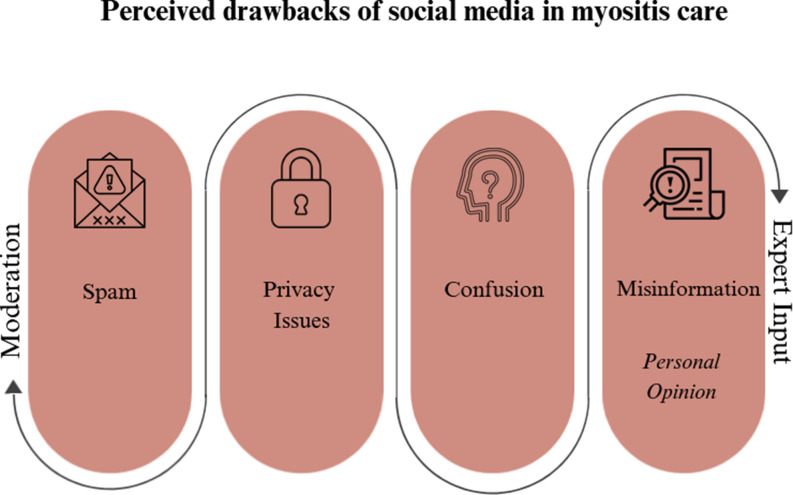
Highlights the drawbacks of social media use for myositis care while presenting possible strategies to address these concerns

While social media provides a platform for peer support and information exchange, patients identified drawbacks (Figure 2). Concerns about misinformation, privacy and the quality of information shared were frequently raised. Members expressed the need for reliable, expert-verified content, as unvetted information can lead to confusion or harm. Additionally, patients highlighted the importance of clearer boundaries between personal opinions and factual medical information, suggesting that improved moderation could enhance trust in these platforms.

One of those was professional involvement on social media dedicated to myositis care. The patients suggested that having an expert presence could enhance these communities by providing reliable information, helping users navigate medical discussions and fostering a better understanding of their conditions.

*‘I would like to see an active physician on each of these pages. Not to give advice*,* of course. It would be interesting for him to see how disempowered people feel when physicians don’t listen. This expert could help find the latest research and post articles to educate users so they’re better aware of what to ask their own doctors. This would really benefit these sites.’ (P 9*,* pos. 51)*.

### Netnography findings

The social media group examined in this netnographic study consists of over 100 members and is moderated by multiple administrators. It has been active for several years and requires prospective members to answer screening questions, which are reviewed before admission. The group operates under a set of established guidelines aimed at fostering constructive discussions, preventing disruptive behavior, and safeguarding member privacy. Engagement levels are high, with posts consistently receiving responses or reactions, indicating an active and supportive community dynamic.

Main topics discussed in the social media group dedicated to myositis care included:

#### Symptom management and treatment

Group members frequently seek advice on managing symptoms such as respiratory issues, skin changes and fatigue. Discussions often revolve around medication, potential side effects and alternative treatments, with members sharing their personal experiences and recommendations. Posts titled *“What worked for me?”* highlight success stories, where individuals share effective coping strategies that have helped them adapt to their condition.

#### Research and information sharing

The group is actively used to disseminate and discuss research published in medical journals such as Rheumatology International. Members share links to studies, articles and personal insights into recent developments in Myositis treatment, distinguishing between credible sources and unverified content. This exchange allows members to stay informed about advancements that may not always be communicated through traditional healthcare channels, reflecting a desire for continuous learning and empowerment.

#### Emotional support and community Building

**Fig. 3:**
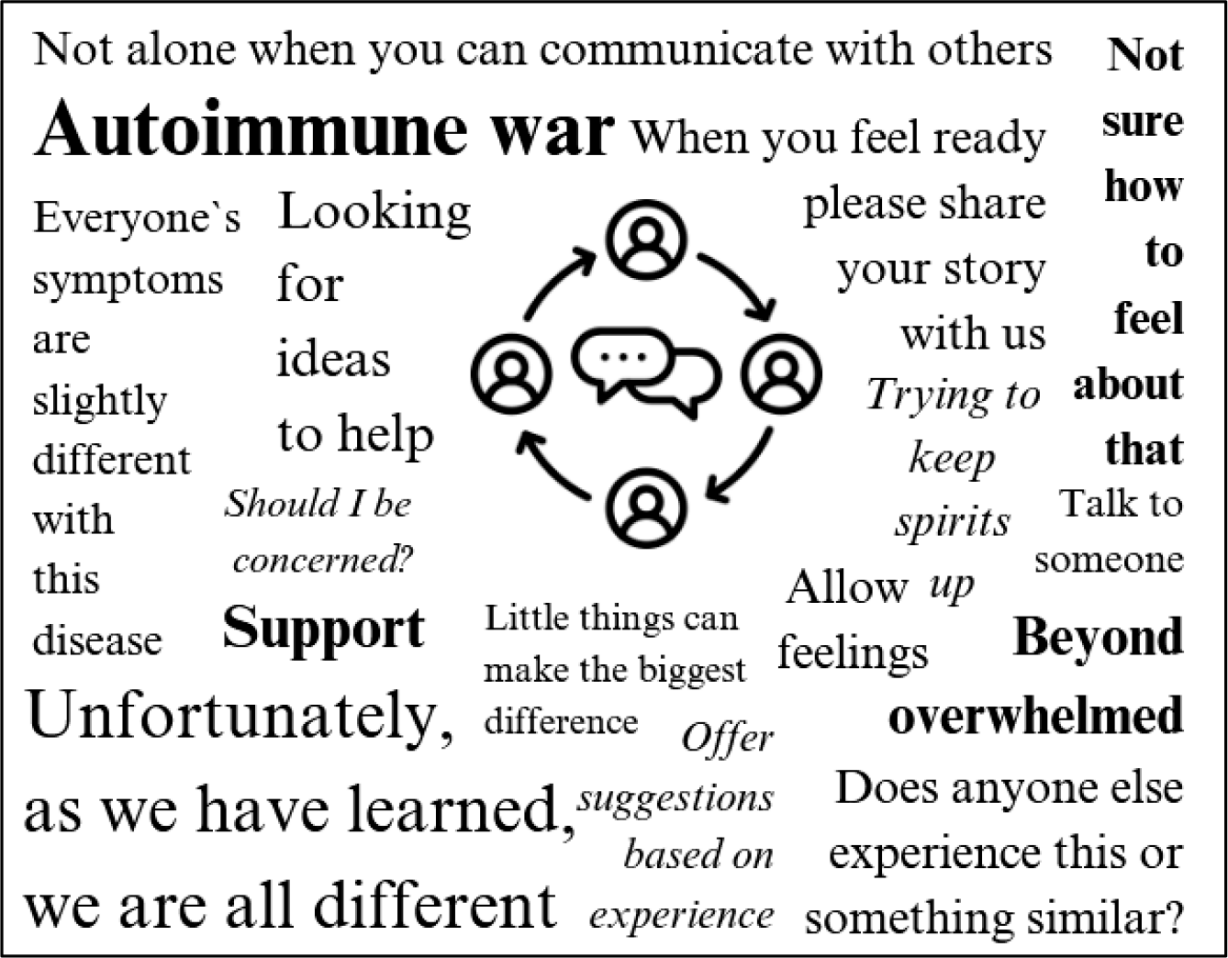
Recurring key words posted in the social media group dedicated to myositis care

Emotional support is a central theme within the group. The tone is predominantly empathetic, warm and encouraging, with members frequently expressing gratitude for the sense of belonging the group provides, emphasizing how valuable it is to share experience with others who face similar challenges. It is highlighted that the group provides a unique sense of connection, helping members feel less isolated and making it easier to process their situation. Recurring key words used within the thread of the social media group were presented word cloud (Fig. 3).

Regular activities, such *as “Smiling Saturday”* encourage members to share positive experiences, fostering a culture of support and positivity. Posts seeking comfort during difficult times often receive numerous supportive responses, including virtual hugs, words of encouragement and shared personal stories. Members describe the group as an environment where they feel able to openly discuss their fears, frustrations, and achievements without facing judgment.

#### Practical advice and self-management

Self-management advice is commonly shared, covering areas such as diet, exercise and mental health strategies. Members exchange ideas on managing day-to-day challenges, ranging from nutritional supplement use to coping with chronic pain and fatigue. The group’s collective knowledge helps individuals make informed decisions about their care and promotes self-efficacy, empowering them to take control of their health journey.

#### Communication with healthcare providers

While direct medical advice is not provided, the group occasionally discusses communication strategies with healthcare providers. Members share experiences of navigating healthcare systems, preparing for appointments and advocating for themselves or their loved ones. The need for improved communication with medical professionals is a recurring topic with suggestions that healthcare providers could benefit from understanding the lived experiences shared within these groups.

### Triangulation of the results: social media in myositis care

The insights from interviews align with active discussions observed in the netnographic data, as presented in Table 2.

**Table 2:** Triangulation of results from interviews and netnography on social media in myositis care

Key Findings	Qualitative Interviews	Netnography	Integrated Analysis
Health Management	Patients emphasized the value of sharing practical advice and experiential knowledge such as coping strategies and symptom management	Posts like “What worked for me?” highlight successful strategies shared by group members for managing symptoms	Social media facilitates the exchange of practical tips and shared experiences, helping patients navigate daily challenges
Emotional Support	Emotional support fosters a sense of belonging and reduces isolation. Peer-to-peer interactions are vital for coping	Group activities like “Smiling Saturday” and supportive comments promote positivity and mutual encouragement	Emotional connections strengthen well-being, create a sense of community, and provide mental resilience for patients
Access to Reliable Information	Challenges in accessing credible information outside social media. Moderation of reliable content is highly valued	Group members actively share research articles and distinguish between credible and unverified sources in discussions	Moderation and sharing of verified content build trust and ensure information reliability within the group
Global Accessibility	Patients emphasised the global, time zone-independent connection that social media provides	The group had members from diverse countries, demonstrating international reach and engagement	Social media bridges geographical and cultural barriers, fostering global collaboration and support among patients
Misinformation	Patients expressed concerns about misinformation and the need for expert-validated content.	Active moderation and rules (e.g., privacy, respect) aim to minimize misinformation while maintaining a safe environment	Addressing misinformation through expert engagement and clear rules strengthens the trustworthiness of online communities

## Discussion

In this study, we explored experiences of patients with myositis and the role of social media within myositis care using qualitative data from interviews and a netnographic analysis in a private social media group dedicated to myositis care.

In the interviews, patients emphasized the heterogeneity of myositis and the diverse range of personal experiences among those affected. Patients confirmed the long diagnostic delays reported in literature [1–3]. Participants emphasised how social media platforms, particularly Facebook groups, are helping to fill gaps in traditional care by providing a global, accessible support network. This community allows patients to share information, get advice on managing their symptoms and provide emotional support, all of which are instrumental in coping with the challenges of living with myositis. Patients particularly value the peer-driven knowledge shared in these groups, which often provides practical insights and lived experiences which complement traditional medical advice. Patients share success stories and practical management strategies, such as dealing with side effects or navigating through healthcare systems, which contribute to a richer understanding of disease management. The experiential knowledge patients receive from each other goes beyond clinical treatment, enabling them to make informed decisions and feel empowered in managing their condition. This aligns with previous research highlighting the importance of peer support in managing chronic diseases [19–21].

Our results also highlight the dual nature of social media, which is both useful and potentially challenging for healthcare support. On the positive side, platforms such as Facebook provide a real-time, accessible medium for patients to connect with others worldwide, alleviating feelings of isolation often associated with rare diseases in a safe and supportive environment. Nonetheless, challenges such as spam, unwanted comments, and concerns regarding misinformation remain. However, the active moderation observed within the netnography seemed to alleviate some of these problems and confirmed the results of earlier studies [20, 22] that indicate that structured moderation in online health communities increases user satisfaction, maintains the quality of discourse, and promotes a sense of support among participants. An important result from this study is the request for a professional involvement within the online community. Some patient support organisations deliberately restrict access to medical professionals to their social media groups, unless they personally have this condition. Where available, they call upon the medical advisory board of the organization, to provide expertise as needed. In some patient-led groups, moderators act as intermediaries, relaying questions from the community and important discussion topics to the medical advisors, who can address them in webinars or other patient-focused educational events. This caters for the different needs of patients and reflects the different approaches of online communities – some value direct expert input to stay informed about medical advances, while others prioritise patient autonomy and privacy and foster a safe space for sharing amongst peers. This is consistent with the findings of other recent research [23], which also concludes that structured moderation and expert participation could address misinformation concerns while still allowing patients to maintain autonomy within these platforms.

Overall, our study shows that social media is a valuable, complementary resource for myositis patients. It provides a community of shared experience, facilitates knowledge sharing, and offers emotional support, all of which are essential aspects of managing chronic, complex diseases. This aligns with recent research demonstrating the critical role of social media in rheumatology [24]. As social media is increasingly integrated into patient care, it is crucial to address privacy concerns and improve the reliability of information in order to optimise these platforms for the benefit of patients. Our study provides new insights by explicitly highlighting how structured moderation in patient-led social media communities effectively mitigates challenges such as misinformation and unwanted interactions. Furthermore, we show how peer-to-peer exchange within these online platforms significantly complements traditional clinical care and improves patients’ self-determination and self-management skills in myositis care.

### Limitations

This study has several limitations. The sampling strategy is prone to selection bias, as we likely interviewed predominantly highly engaged myositis patients who are active on social media. This may have restricted our ability to capture the full range of patient experiences, particularly those of individuals who are less engaged online or do not participate in social media or support groups. Additionally, the focus of our netnographic analysis on a single social media group dedicated to a specific subset of myositis patients limits the transferability of our findings to other online communities or platforms. While other social media platforms may play a significant role in myositis care, they were not prominently reflected in our data. Future research should further explore why platforms such as Instagram, X (formerly Twitter), or YouTube were less frequently used by patients with myositis. Another limitation of this study is the gender distribution of participants, as the majority of interviewees were women. As a result, the findings may not fully capture all perspectives. Future studies, particularly quantitative ones, could provide a broader understanding of how social media usage varies across the myositis community and different platforms. Finally, our findings predominantly highlight the positive aspects of social media in myositis care. However, further research is needed to explore potential negative effects, such as increased risk of depression, anxiety, and stress associated with social media use [25]. Interestingly, participants did not report adverse individual consequences of social media use. While we hypothesize that there may be negative psychological effects, these might need to be explored using other methodological approaches (e.g., not self-reported).

## Conclusion

Social media serves as a pivotal tool for individuals living with myositis, enabling information exchange, peer support, and guidance on self-management. These platforms foster knowledge sharing and emotional support, addressing gaps often present in traditional healthcare systems. However, significant challenges persist, including concerns about data privacy, exposure to unwanted or distressing content, and the spread of unverified information. The findings of this study underscore the importance of raising awareness of the impact of social media resources, incorporating social media into holistic care strategies, and enabling social media to complement traditional healthcare approaches to better support patients with myositis.

## Electronic supplementary material

Below is the link to the electronic supplementary material.

Supplementary Material 1

Supplementary Material 2

Supplementary Material 3

Supplementary Material 4

Supplementary Material 5

## Data Availability

The datasets used and/or analysed during the current study are available from the corresponding author on reasonable request. All data relevant to the study are included in the article or uploaded as supplementary material. For further questions regarding the reuse of data, please contact the corresponding author (KB).

## Declarations

### Disclaimer

No part of this manuscript, including text and graphics, has been copied or published elsewhere, either in whole or in part. No AI was used to generate original research content or results. The views and opinions expressed are solely those of the author and do not represent or reflect those of any affiliated institution (LG).
