# Supplementary material for: Social media in myositis care – an exploratory mixed-methods study among myositis patients (SociMyo)
Source: Rheumatol Int. 2025 Jun 4;45(6):149. doi: 10.1007/s00296-025-05903-6 (PMC12137372; doi:10.1007/s00296-025-05903-6)
Supplement: Supplementary file 2 — Supplementary Material 2 [file 296_2025_5903_MOESM2_ESM.docx]

| **Introduction:**  Thank you for participating in this interview about social media use in Myositis care. The purpose of this interview is to gain insights into how social media platforms are being utilized by individuals with Myositis to seek support, share information, and engage with the community. The information you provide will help us better understand the impact of social media on Myositis care and potentially improve the support available to those affected. Please note that your responses will be kept confidential, and you may choose to skip any question you do not wish to answer. With that in mind, let's get started! |  |
| --- | --- |
| **1. Background and Social Media Usage:**  a. Can you briefly introduce yourself and tell us about your experience with Myositis?  b. How long have you been using social media platforms?  c. Which social media platforms do you actively use, and how frequently do you engage with them?  d. What motivates you to use social media in the context of Myositis care? Are there any specific goals you hope to achieve through these platforms? | **Follow-up questions:**  Can you tell us more about this?  And then?  How was that for you?  How do you see that?  Can you please go into this in more detail?  Could you please give an example?    What do you mean in concrete terms?  Can you tell us more about this? |
| **2. Information Seeking and Knowledge Sharing:**  a. How do you typically use social media to seek information about Myositis care? Do you follow specific pages, groups, or hashtags?  b. Have you found social media to be a valuable source of information about Myositis? Why or why not?  c. Do you actively participate in discussions or share your own experiences related to Myositis care on social media? If so, what prompts you to engage with others? |  |
| **3. Support and Community Engagement:**  a. Have you joined any Myositis-related online communities or support groups on social media platforms? If yes, what are the benefits you derive from such communities?  b. How has social media helped you connect with others who have Myositis? Do you find it comforting or empowering to interact with individuals who share similar experiences?  c. Can you share any positive or negative experiences you've had while engaging with the Myositis community on social media platforms? |  |
| **4. Privacy and Data Security:**  a. Are you concerned about your privacy and data security when using social media platforms for Myositis-related interactions? Why or why not?  b. What measures do you take to protect your privacy while engaging on social media? |  |
| **5. Professional Guidance and Credibility:**  a. Do you encounter healthcare professionals or organizations on social media who provide Myositis-related guidance or information? How do you assess the credibility of such sources?  b. Are there any challenges or concerns you have regarding the accuracy or reliability of information shared by healthcare professionals or organizations on social media? |  |
| **6. Recommendations and Suggestions:**  a. Based on your experiences, what improvements or additional features would you like to see in social media platforms to better support individuals with Myositis?  b. Are there any specific guidelines or ethical considerations you believe social media platforms should follow to ensure a safe and supportive environment for Myositis-related discussions? |  |
| **Conclusion:**  Thank you for sharing your insights and experiences regarding social media use in Myositis care. Your input will contribute to a better understanding of how these platforms impact the lives of individuals with Myositis. Is there anything else you would like to add before we conclude the interview? |  |
