# Supplementary figures and images for: Social media in myositis care – an exploratory mixed-methods study among myositis patients (SociMyo)
Source: Rheumatol Int. 2025 Jun 4;45(6):149. doi: 10.1007/s00296-025-05903-6 (PMC12137372; doi:10.1007/s00296-025-05903-6)

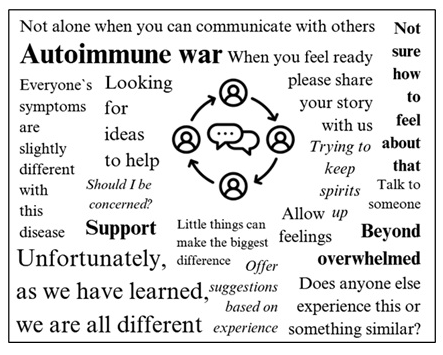

Supplement: Supplementary file 3 — Supplementary Material 3 [file 296_2025_5903_MOESM3_ESM.png]
