# Supplementary material for: Social media in myositis care – an exploratory mixed-methods study among myositis patients (SociMyo)
Source: Rheumatol Int. 2025 Jun 4;45(6):149. doi: 10.1007/s00296-025-05903-6 (PMC12137372; doi:10.1007/s00296-025-05903-6)
Supplement: Supplementary file 4 — Supplementary Material 4 [file 296_2025_5903_MOESM4_ESM.pdf]

# ***Rheumatology*** Author Disclosure Statement

**MANUSCRIPT INFORMATION**

Manuscript Number: RHE- RHEI-D-25-00256

Manuscript Title: Social media in myositis care – an exploratory mixed-methods study among myositis

Corresponding author: Katharina Boy

**The purpose of this form is to provide the readers of *Rheumatology* with information regarding any interest and relationships that may influence how they read and understand your work. It also serves to confirm that all individuals qualify for authorship as set out in the form below. The form should be completed by the corresponding author on behalf of all authors. It is the corresponding author's responsibility to ensure all authors' potential conflicts of interest are accurately reported.**

## Section 1: AUTHORSHIP

As corresponding author, I certify:

- All authors have made a substantial contribution to the conception and design, or the acquisition of data, or the analysis and interpretation of data, as well as to the drafting or critical revision of the manuscript, and accept public responsibility for portions of the content.
- All authors have seen and agree with the order of authorship as stated on the accepted manuscript and verify that all persons named as authors meet the criteria for authorship.
- All authors verify that the manuscript represents valid work.
- All authors have reviewed the final manuscript and approve it for publication.
- All authors confirm that neither this manuscript, nor any other with substantially similar content by one or more of the same authors, has been published, accepted or is currently being considered for publication elsewhere except as an abstract.
- I confirm that all authors have had access to the raw data and that, upon request, will produce the data on which the manuscript is based for examination by the Editor or their assignee.
- All non-authors who have made substantial contributions to the work reported in the manuscript (including writing and editing assistance) are named in the acknowledgements and have given permission to be named.

☑ **By checking this box I, the corresponding author of this manuscript, certify the above to be true.**

## Section 2: CONFLICT OF INTEREST

**This section of the form concerns any interests the authors may have that could may influence how the work conducted and reported is perceived. It is the corresponding author's responsibility to confirm any interests listed for any author in the paper are still current and provide the Editorial Office with clarification should there be changes to any authors' interests after a paper is submitted.**

All authors are required to disclose any direct or indirect interests (dual commitments), financial or otherwise, that might affect or be perceived to affect the conduct or reporting of the work they have submitted. For the purposes of this form we wish only to know about relationships, current or in the past **three years**, that are relevant to the manuscript being published, as well as relationships, past or present, that have direct relevance to materials used in the study.

Such financial interests may take the form of corporate appointments, consultancies, shareholding, or other equity interest or patent licensing arrangements with companies mentioned in or related to the subject matter of the article being submitted. If the authors are uncertain about what might be a dual commitment, they should err on the side of full disclosure. Any limitation to full access to all material must be disclosed. This is particularly important for any manuscripts detailing work supported in part or entirely by a pharmaceutical or instruments manufacturer/supplier.

**Please select either A or B. If you select B please provide details in the box below.**

◉ **A.** I declare that all authors have no conflicts of interest.

○ **B.** I hereby disclose the following authors have financial interest, direct or indirect (dual commitment) that might be perceived as affecting the conduct or reporting of the work submitted:

These interests are in the form of:

- ☐ Corporate appointments
- ☐ Other equity interests
- ☐ Member of speakers' bureau
- ☐ Consultancies
- ☐ Received royalties
- ☐ Received grants/research support
- ☐ Shareholder/stock ownership
- ☐ Received honoraria
- ☐ Received other financial/material support

The disclosure statement as it appears in the manuscript is as follows:

KB, NO, GN, LW, JK, MH and LG declare no conflicts of interest.
SM and FM have received funding from AbbVie and Novartis, unrelated to Myositis.
KC is an employee of Sobi, working on projects unrelated to this manuscript.
LG is co-chair of EMEUNET social media and serves as social media editor for the Journal of Clinical Rheumatology.

☑ **By checking this box, on behalf of all authors, I confirm that all authors have been consulted prior to completing this form and comply with the requirements for authorship (section 1) and have declared any potential conflicts of interest (section 2).**

Full Name: Katharina Boy

Date: 18.02.2025

Corresponding author e-mail address: katharina.boy@mhb-fontane.de

**This form should be completed by the corresponding author and returned to the Editorial Office at editorial@rheumatology.org.uk.**

**Submit to RHEUMATOLOGY**
